# Comparison of clinical knowledge management capabilities of commercially-available and leading internally-developed electronic health records

**DOI:** 10.1186/1472-6947-11-13

**Published:** 2011-02-17

**Authors:** Dean F Sittig, Adam Wright, Seth Meltzer, Linas Simonaitis, R Scott Evans, W Paul Nichol, Joan S Ash, Blackford Middleton

**Affiliations:** 1 UTHealth-Memorial Hermann Center for Healthcare Quality & Safety, School of Biomedical Informatics, University of Texas Health Science Center, Houston, TX, USA; 2 Department of Medicine, Brigham & Women's Hospital, Harvard Medical School, Boston, MA, USA; 3 Clinical Informatics Research & Development, Partners Healthcare System, Boston, MA, USA; 4 Regenstrief Institute, Inc.; Indiana University School of Medicine, Indianapolis, IN, USA; 5 Department of Medical Informatics, Intermountain Health Care, Department of Biomedical Informatics, University of Utah, Salt Lake City, UT, USA; 6 Patient Care Services, Veterans Health Administration, Seattle WA, USA; 7 Department of Medical Informatics, Oregon Health & Science University, Portland, OR, USA

## Abstract

**Background:**

We have carried out an extensive qualitative research program focused on the barriers and facilitators to successful adoption and use of various features of advanced, state-of-the-art electronic health records (EHRs) within large, academic, teaching facilities with long-standing EHR research and development programs. We have recently begun investigating smaller, community hospitals and out-patient clinics that rely on commercially-available EHRs. We sought to assess whether the current generation of commercially-available EHRs are capable of providing the clinical knowledge management features, functions, tools, and techniques required to deliver and maintain the clinical decision support (CDS) interventions required to support the recently defined "meaningful use" criteria.

**Methods:**

We developed and fielded a 17-question survey to representatives from nine commercially available EHR vendors and four leading internally developed EHRs. The first part of the survey asked basic questions about the vendor's EHR. The second part asked specifically about the CDS-related system tools and capabilities that each vendor provides. The final section asked about clinical content.

**Results:**

All of the vendors and institutions have multiple modules capable of providing clinical decision support interventions to clinicians. The majority of the systems were capable of performing almost all of the key knowledge management functions we identified.

**Conclusion:**

If these well-designed commercially-available systems are coupled with the other key socio-technical concepts required for safe and effective EHR implementation and use, and organizations have access to implementable clinical knowledge, we expect that the transformation of the healthcare enterprise that so many have predicted, is achievable using commercially-available, state-of-the-art EHRs.

## Background

Following passage of the American Recovery and Reinvestment Act of 2009 [1], which provides significant financial incentives over 5 years to eligible hospitals and physicians that implement certified electronic health records (EHR) with clinical decision support (CDS), defined as providing appropriate information to help guide medical decisions at the time and place of care, the pressure on commercial EHR vendors to deliver full-featured, easy to use systems has increased significantly. Several recent studies have suggested that EHRs without advanced clinical decision support (CDS) features and functions may not provide the promised improvements in patient safety or quality of care [2,3].

Over the past several years, we have carried out an extensive qualitative research program focusing on the barriers and facilitators to successful adoption and use of advanced, state-of-the-art clinical information systems [4,5]. Much of our early work focused on large, academic, teaching facilities with long-standing programs in clinical information system research and development [6]. Over the past several years, we have begun investigating community hospitals and clinics that rely on commercially-available EHR systems [7]. Over these many years, we have seen many different types of EHR systems implemented in many different setting using many different techniques. A result of this work has been development of a new 8-dimension, socio-technical model that we believe helps explain why some implementations go well and others poorly [8]. Clearly, the quality of the technology (i.e., the first dimension of the model), as measured by the features and functions that are available to end-users, plays a major role in any successful implementation. In an attempt to better quantify our qualitative findings that almost all of the EHR systems that we have investigated have nearly the same basic features and functions, as evidenced by the fact that all of them have been CCHIT-approved, we decided to survey a select group of commercial EHR vendors regarding their clinical decision support and knowledge management capabilities.

In order to provide a reference context for the current extent of CDS capabilities, we compared the survey responses of nine commercial vendors with those of four leading internally-developed non-commercial systems. Chaudhry et al. found that approximately 25% of all English-language, peer-reviewed studies that have been used to demonstrate the increases in quality and safety of patient care originated from only four U.S.A healthcare institutions (Intermountain Health Care, Salt Lake City, UT; Veterans Health Administration, Washington, D.C.; Regenstrief Institute, Indianapolis, IN; Brigham & Women's Hospital, Boston, MA). Furthermore, these four institutions provided the majority (54 of 76) of the high-quality literature Chaudhry identified in this area [9]. Chaudhry concluded that studies from these institutions demonstrate that "a multifunctional system can yield real benefits in terms of increased delivery of care based on guidelines (particularly in the domain of preventive health), enhanced monitoring and surveillance activities, reduction of medication errors, and decreased rates of utilization for potentially redundant or inappropriate care." However, they noted that the methods and systems used by these pioneering organizations to obtain these benefits required iterative refinement of locally-developed systems over many years. They, as well as others, have concluded that such long-term, resource intensive, internal health information technology development projects are not a viable option for institutions interested in participating in the recent HITECH stimulus funding which requires significant functionality be in widespread use by 2012 [10].

Therefore, we sought to assess whether the current generation of commercially-available EHRs are capable of providing the technical infrastructure required to deliver and maintain the clinical decision support interventions required to support the recently defined "meaningful use" criteria [11].

## Methods

### Survey development

We developed a 17-question survey to assess availability of various clinical knowledge management features, functions, tools, and techniques provided by EHRs currently in use. By "clinical knowledge management", we mean the entire process by which clinical knowledge is created, made available, and maintained within an EHR system. This includes the software tools necessary to organize and define knowledge, along with the organizational procedures necessary to manage this knowledge. At the highest level, this requires a systematic and consistent approach to update and maintain the comprehensive set of clinical decision support functions - when a manual ad hoc approach is no longer feasible. A key assumption of this work is that the clinical knowledge management features and functions these systems provide are independent of whether the EHR is designed for the in-patient or out-patient clinical environment. This survey is a quantitative extension of an on-going multi-site, qualitative research project designed to study facilitators and barriers to successful creation, adoption, and dissemination of advanced, clinical decision support to healthcare organizations of all types across the nation [12]. The survey was further informed by the evolving "meaningful use" definition. Finally, the questions were field tested and refined during multiple in-person interviews and demonstrations of existing functionality with EHR vendors at a national EHR vendor conference.

### Survey description

The survey was divided into three parts. The first asked basic questions about the electronic health record (EHR) product. For example, we were interested in knowing the name and version of their current product, the different modules (e.g., computer-based provider order entry (CPOE), results review, clinical documentation, etc.) in their system that were capable of providing clinical decision support, how many clinicians were currently using their system nationwide, and what their EHR delivery model was (e.g., on-site server or via a remote hosting solution). This section was designed to assess the capability of the current generation of EHRs to deliver and manage various CDS interventions.

The second part of the survey asked specific questions about the clinical decision support-related system tools and capabilities that each vendor provides. We asked the following questions (the information in italics was designed to clarify each question):

• Does your system allow the collection of structured data within clinician notes [13]? *Structured data can be collected via drop-down lists, from nested menus, or by matching typed free text to a controlled clinical vocabulary. This structured data is then theoretically available for use by the CDS logic*. We asked those that responded affirmatively, "what controlled vocabularies they use to record the structured clinical findings (e.g., Medications, Laboratory tests, Allergies, Problems/Diagnoses, Procedures)?"

• Does your system support ad-hoc queries (registries) of patient data by end-user [14]? *These queries could be used to support population management, e.g. to identify a group of similar patients taking a medication that was recently recalled, or in need of a preventive health screening exam*. If yes, we asked, "Do you have a replicated database? And "How often is it updated?"

• Do you support the HL-7 InfoButton standard [15]? *The InfoButton provides a mechanism for the user to click on a "hyperlink" from within the EHR and be taken to a relevant place in an online information resource (e.g., Micromedex or CliniGuide)*.

• Does your product support the Arden Syntax for Medical Logic Modules (MLMs) [16]? *MLMs are used to share clinical decision support logic that is most often used to create real-time, point-of-care clinical decision support alerts*.

• Does your system provide a clinical knowledge editor so end-users can modify or create their own clinical decision support interventions [17]? *This editor would enable one of your customers to create "if-then" logic. The editor should help them identify specific coded data items within the EHR's database (e.g., patient_Date_of_Birth, or Weight) and then apply various mathematical (e.g., Greater than) or Boolean (e.g., AND, OR, NOT) operations and if true send a message or alert to the clinician (e.g., elderly patients should not take valium)*.

• Do you have a clinical content management system separate from the EHR that allows users to see when their content should be reviewed/updated [18]? *This content management system would allow end-users to browse, sort, or filter the clinical content (a.k.a., the clinical knowledge) that is used within the EHR to create the CDS. In addition, the content management system should contain some "metadata" that further describes the clinical content (e.g., date it was created, author, clinical condition it treats, where it is used in the EHR, etc.). Users should also be able to search or filter content based on this metadata*.

• Do have a reporting capability that allows healthcare organizations to track the effect and/or usage of the CDS content [10]? (Can you measure the response rate for alerts, or order set usage?) *For example, if you had a rule to remind clinicians to order Hemoglobin A1c (HbA1c) tests on all diabetics at least every year, then you would like a reporting capability that could calculate the percentage of all diabetics who actually got an HbA1c test in the past year. Likewise, you might like to know how often this HbA1c reminder has fired in the last year and what percentage of the time clinicians "accepted" or followed this reminder*.

The final section of the survey asked questions about Clinical Content. Specific questions included:

• How is the CDS content implemented and maintained (e.g., customer is responsible for configuring and maintaining all content to...vendor maintains all content from a central location along with the EHR database)? *We are particularly interested in where the CDS logic/content resides, who maintains and updates the logic, and the processes that are involved in these updates*.

• Who is your medication drug database supplier [20]?

• Do you support any other third party CDS content vendors (e.g. for order sets or alerts) [21]?

• Do you provide clinical content (alert logic, order sets, condition-specific displays) to your clients [22]? *Some vendors call this a "starter-set" or model system that they expect the client to modify before using in their production system. Some vendors provide their clients with charting templates, condition-specific data displays, and even alerts they can use*. If yes, we asked, "what kind of content? How much do you provide, and how and how often do you update this clinical content?

• Do you support an on-line collaborative knowledge development environment (perhaps using SharePoint or other Web 2.0 technologies) that allows customers to collaborate asynchronously on CDS content [23]?

• Do you have an Internet accessible repository for clients to share their locally generated clinical decision support content with other clients [24]?

### Site selection

We selected nine commercially available EHR vendors (three inpatient; six outpatient) from the 2008 list of Certification Commission on Health Information Technology (CCHIT) approved EHR vendors [25]. The vendors were selected because of their size, market position and willingness to cooperate with research. We selected the top four institutions (all of which combined inpatient and outpatient systems) that were responsible for generating approximately 25% of the citations identified by Chaudhry et al. (2006) [9].

### Survey distribution

After receiving Institutional Review Board (IRB) approval from the University of Texas (HSC-SHIS-09-0323), we sent the survey via email to representatives (e.g., product managers or Chief Medical Information Officers) of the nine EHR vendors as well as to colleagues at each of the four institutions with internally-developed systems. In several instances we followed up our email with a teleconference to clarify one or more of our questions, their responses, or both.

### Data analysis

All responses were collated, summarized, and are presented anonymously (as agreed upon by all vendors).

## Results

Tables 1 and 2 provide information on the EHR vendors and institutions we surveyed along with the location of their headquarters, current version, and number of clinical users of their system.

**Table 1:** Overview of vendors surveyed

Vendor	Version	# Users
**Allscripts, Chicago, IL**	Professional EHR v8	60,000

**Cerner Kansas City, MO**	Millennium 2007.19	13,000

**Eclipsys, Atlanta GA**	Sunrise Clinical Manager v5.0sp4	25,000

**e-MDs, Austin, TX**	Solution Series v6.3	6,000

**Epic Systems Verona, WI**	Inpatient Summer '09	160,000

**GE Healthcare Wauwatosa, WI**	Centricity v9.5	"Thousands"

**Greenway Medical Technology Carrollton, GA**	PrimeSuite 2008	4,000

**NextGen, Horsham, PA**	EMR v5.5.27	45,000

**Spring Medical Systems Houston, TX**	SpringCharts EHR v9.7.5	1,500

**Table 2:** Overview of internally-developed Systems surveyed

Institution	Version	# Users
**Intermountain Health Care Salt Lake City, UT**	HELP1 and HELP2	20,000

**Partners HealthCare Boston, MA**	Longitudinal Medical Record (Outpatient) Brigham Integrated Computer System (Inpatient)	32,515

**Regenstrief Institute Indianapolis, IN**	Gopher CPOE System (Inpatient and Outpatient)	2,500 clinicians entering orders

**Veterans Health Administration, Washington, DC**	VistA: Computerized Patient Record System V1.0.27.90	172,000

Table 3 presents the EHR vendor's responses to all of the clinical decision support and knowledge management questions we posed. Vendors requested their identities remain anonymous, therefore we have randomly ordered the columns of table 3. Table 4 presents a similar summary of the responses to all questions from the institutions with the internally-developed systems.

**Table 3:** Vendor responses to clinical decision support and knowledge management questions

Questions	EHR: 1	EHR: 2	EHR: 3	EHR: 4	EHR: 5	EHR: 6	EHR: 7	EHR: 8	EHR: 9
**CIS modules with CDS?**	Y	Y	Y	Y	Y	Y	Y	Y	Y

**EHR delivery model?**	Onsite or Remote host	Onsite or Remote host	Onsite or Remote host	Onsite or Remote host	On site only	Onsite or Remote host	Onsite or Remote host	Onsite or Remote host	Onsite or Remote host

**CDS content implemented, configured, maintained by customer or vendor?**	Customer	Vendor	Vendor	Vendor	Vendor	Customer	Vendor	Customer	Vendor

**Collect structured data - controlled vocabularies?**	Y - multiple	Y - multiple	Y - multiple	Y - multiple	Y - multiple	Y - multiple	Y - multiple	Y - multiple	Y - multiple

**Support ad-hoc queries? Live replicating database?**	Ye -live db	Yes - db replicated	Yes. No db	Yes.	Yes - Yes	Yes - Yes	Yes - daily update db	Report library - no	Yes-db replicated

**HL7 InfoButton?**	Yes	Nonstandard	No	No	Nonstandard	Yes	No	Nonstandard	Yes

**Arden Syntax for MLM?**	Yes	No	No	No	Nonstandard	No	No	Yes	Yes

**Clinical knowledge editor for user to create CDS?**	Yes	No	Yes	No	Yes	Yes	Yes	Yes	Yes

**Content mgmt system separate from EHR?**	Yes	No	Yes	No	Yes	No	No	No	Yes

**Reporting capability to track effect/usage of CDS?**	Yes	Yes.	Yes	No	Yes	Yes	No	N/A	Yes

**Drug database supplier?**	Multum, FDB	FDB	Proprietary and FDB.	Medi- Span and FDB	FDB, Medi-Span, Lexicomp, Micromedex	FDB	FDB	FDB	Multum MediSource

**3rd party CDS content vendors?**	Zynx	Multiple	No	No	Multiple	No	No	No	Zynx

**Provide clinical content (starter sets). How often and is it updated?**	Y- client community	Y- via web	Y-via web	Yes-irregular	Y-1x/year	Y- irregular	Y - irregular	Y-biweekly via web	Y-1x/year

**On-line collaborative knowledge development environment (web 2.0)?**	Yes, SharePoint	No, listserv	Yes	No	Yes	Yes	Yes	No, listserv	Yes

**Internet repository for clients to share CDS?**	Yes	No	Yes	No	Yes	Yes	Yes	No	Yes

**Table 4:** Responses from the internally-developed EHR systems

Questions	Partners	Regenstrief	Intermountain Health Care	Veterans' Health Administration
**CIS modules offered (products that have capability to provide decision support)?**	Yes, CPOE, results review, laboratory, outpatient clinical documentation, eMAR, Drug-Drug/Allergy/Lab Interaction checking. BCMA	Yes, CPOE, consults from specialists. Documentation: patient visit notes, ADT summaries, problem lists. Links to test results, notes	Yes, Antibiotic, TPN; Results review; RN, RT, Pharmacist charting; Infection & Disease monitoring, ADR triggers, VTE high risk alerts	Yes, Order menus, sets, quick orders. documentation. Templates. Drug-Drug/Allergy/Lab Interaction checking. BCMA

**EHR delivery model?**	Onsite	Onsite	Onsite (Server and web based)	Onsite (move to regional server)

**CDS content implemented/configured/maintained by customer or vendor?**	maintained by informaticians. Most content separate from code viewed thru repository	Clinician/Informaticians update terminology and decision support rules as needed	Users responsible for content. Programmers logic. Updated weekly, larger updates quarterly	CDS maintained, implemented locally. National releases with local configuration.

**Collect structured data and which controlled vocabularies?**	LOINC, RxNorm, SNOMED, ICD-9, CPT, FDB	ICD-9 & CPT codes avail. Most entered via Regenstrief Dict. mapped to standard vocabs.	Mostly coded data, some dictated notes as free text. LOINC, SNOMED, ICD-9	Not from clinician notes, but reminder templates do. ICD-9 for problem list

**Support ad-hoc queries? Live replicating database?**	Yes. Yes, weekly to monthly depending on data element	Queries done by data analysts on real database, in response to requests from end users.	Yes, but most reports run on EDW from programmers. Have some canned reports monthly	Yes, use replicated DB, varied update frequency; ProClarity data cubes

**HL7 InfoButton?**	Yes	No	Yes	No, but something similar

**Arden Syntax for MLM?**	No	No. Gopher Care Rules corresponds to Arden Syntax.	No (some graduate projects)	No

**Clinical knowledge editor for user to create CDS?**	Yes	Editor used by infomaticians to create CDS	Ability for knowledgeable users to create CDS	Not by end user. Some local configuration by support staff

**Content mgmt system separate from EHR?**	Yes	No	Online K repository, most review/updates programmers	No. Pilots are ongoing.

**Reporting capability to track effect/usage of CDS content?**	Yes	User responses prospectively, not retrospective	Yes, from reports in the EDW	Yes, focused on performance measures not CDS usage

**Drug database supplier?**	FDB	In-house drug terms	FDB	Internal Drug/Allergy files

**3rd party CDS content vendors?**	Thompson Micromedex, UpToDate	No	MicroMedex through Info-buttons	MicroMedex, UpToDate, etc. at point of care

**Provide clinical content (starter sets). What content is included, how and how often and is it updated?**	CDS content provided and maintained by the Informatics groups. Updates distributed on an as needed basis.	Alerts, drug-drug. Formulary, price. Order sets. Default drug strengths, frequencies, or instructions. updates mult/yr.	All alert logic is provided, developed, and maintained by institution. weekly and quarterly updates	Local development of reminders, order menus, document templates.

**On-line collaborative knowledge development environment web 2.0?**	Yes	No	No	Yes, starting SharePoint sites for national groups

**Internet repository for clients to share locally generated CDS?**	No	No	No	Some networks have web sites to share CDS

### Summary of key findings

All of the vendors and institutions have multiple modules capable of providing clinical decision support interventions to clinicians. All but one of the vendors offer their products remotely (i.e., via Application Service Provider (ASP) models) as well as via on-site servers. In contrast none of the leading internally-developed systems had this capability, although several of them did allow remote access via browsers.

All but two of the vendors and all of the internally-developed systems allow their clients to control the CDS configuration and updating process, although most vendors bundle the clinical content updates with software upgrades. All of the vendors and internally-developed systems stated that they supported the use of a variety of controlled clinical terminologies (e.g., LOINC, SNOMED, ICD-9, Medcin, RxNorm, etc.) to record structured clinical findings, although not all clients make use of these features. All of the vendors and internally-developed systems provided their customers with the ability to perform *ad hoc *queries or patient registry functionality, although only 5/9 vendors and 2/4 internally-developed systems has a real-time, replicated database for these queries. Only 3/9 vendors and 1/4 internally-developed systems support the HL-7 InfoButton standard, although three other vendors and all the other internally-developed systems have similar, although non-standard, InfoButton-like functionality. Similarly, 3/9 vendors but none of the four internally-developed systems support the Arden Standard for representing medical knowledge.

Seven of the nine vendors and 2/4 of the internally-developed systems provide their customers with a knowledge editor that allows them to create clinical decision support interventions but only 4/9 vendors and 1/4 internally-developed systems store their content in a content management system separate from the application code. Six of the nine vendors and all of the internally-developed systems have the ability to report on the effectiveness of the CDS interventions they deliver, but only 5/9 vendors can report usage statistics of the CDS itself (e.g., alert override rate, or # of times a specific order set was used) whereas all of the internally-developed systems have this capability. All but one of the surveyed vendors and 2/4 of the internally-developed systems reported that they supported First DataBank as their medication-related knowledge base supplier, while 3/9 vendors and 0/4 internally-developed systems reported allowing their customers to utilize multiple commercial medication knowledge base suppliers. Four of the nine vendors and 3/4 of the internally-developed systems reported being able to utilize (i.e., either incorporate into their systems or access via the internet) CDS content from other commercial vendors (e.g., Zynx, Micromedex, UpToDate, etc.). All of the vendors and the internally-developed systems provided their customers with a "starter set" of clinical content (e.g., alerts, order sets, documentation templates, etc.) and at least irregular updates and 6/9 vendors and 2/4 internally-developed systems provide websites (using web 2.0-type functionality) that allow users to share content with others.

## Discussion

The vendors in our sample represent a large cross-section of EHRs used in the United States and internationally. We believe that they are a generally representative sample of knowledge management capabilities of EHRs, with the possible exception that smaller and niche vendors are not well represented.

Based on the report by Chaudhry, as well as prevailing attitudes in the academic medical informatics community, we expected a large gap in the technical capabilities between the four "best-in-class", internally-developed systems as depicted in the scientific literature and commercial EHR vendor's products. We were pleasantly surprised to discover that all of the vendors we surveyed acknowledged and appreciated the challenges of clinical decision support, and reported that they provided knowledge management capabilities to help their customers manage their clinical knowledge. In fact, with respect to the clinical knowledge management capabilities we surveyed, there appears to be little discernable difference between the systems provided by the surveyed EHR vendors and the internally-developed systems. These results mirror our findings from two recent studies that examined the CDS capabilities of commercially-available clinical information systems [26,27]. The vast majority of commercially-available EHR systems currently on the market in the U.S.A. today report that they provide an adequate technical infrastructure to support the required CDS and knowledge management capabilities.

Despite the strong technical capabilities provided by vendors, however, previous research has shown that simply providing healthcare organizations with CCHIT-compliant, CDS and KM feature-filled electronic health record applications will not ensure that every organization will be able to realize the process-level improvements in healthcare quality and patient safety that are possible [28].

First, to achieve maximum benefit, organizations will need to select, implement, and maintain the necessary hardware, networking, and operating system software. Without a robust computing infrastructure, the CDS and knowledge management features and functions will not be able to keep up with end-users demands for sub-second response time and 24 hours a day, 7 days a week availability.

Second, organizations will need to develop or purchase, implement, and maintain the clinical content required to provide the clinical decision support interventions required to help guide clinicians. Managing this content is a critical job of either the end-user, or the end-user in conjunction with the vendor, using the knowledge management tools identified in this survey. Ideally, the individuals involved have informatics training (as described below).

Third, EHR vendors will also have to develop easy to learn and use user interfaces that allow users to interact with the CDS interventions in a seamless manner, in the course of the clinical workflow. Failure to integrate the CDS interventions into the clinicians' routine workflow will undoubtedly result in less than optimal usage. Perhaps one of the main reasons that these internally-developed systems have achieved such great success with their CDS interventions is the fact that all of them have been iteratively refined over many years to more closely approximate the clinical workflow of their users. Another reason could be the sense of ownership and responsibility these developers have to make sure the CDS applications are implemented and maintained.

Fourth, to develop, implement, and maintain all of the required clinical content, new kinds of employees capable of interacting with the information technology and clinical personnel will be required. These clinical "informaticians" will be key in the successful CDS implementation and maintenance efforts. Once again, all of the internally-developed systems were designed, developed, and maintained by such a group of committed, highly-trained clinical informaticians. In addition to managing the clinical content, these informaticians also help redesign the clinicians' workflows to make optimal use of the CDS interventions. Further, clinical practice guideline authors, should take note of the clinical content needs of EMRs for CDS, and aim to deliver guidelines in an unambiguous form as near ready for implementation as possible [29].

Fifth, on top of all these other challenges, the organization must review and modify their clinical workflows and communication patterns [30] along with their internal policies and procedures to reflect newfound capabilities of CDS-enabled EHRs. Failure to make these modifications can result in significantly less then optimal usage of these systems.

Six, the organization must develop the technical and governance infrastructure required to capture, report on, and evaluate the data necessary to measure and monitor both the inner workings of the CDS as well as the effect of the interventions on clinical processes and patient outcomes [31]. Without such careful evaluation, organizations might inadvertently negatively affect the patients' care that they are working to improve [32].

Finally, the local and national bodies responsible for creating and maintaining the rules and regulations that govern the operation of the healthcare facilities and their workers must be updated to take into account the new capabilities of these CDS-enabled EHR systems [33].

### Study limitations

The EHR vendor data presented represents only a small sample of the 2008 CCHIT-approved, commercially available EHR systems. Second, the use of self-reported data from the vendors and the internally-developed systems may result in an overstating or an understating of the current capabilities of their systems. When possible we have spoken directly with customers who have installed and are using these systems, as well as, asking for demonstrations of the systems. To date we have not encountered any "inflation" of capabilities. Finally, even though many of these vendors reported a robust suite of CDS and knowledge management features, our experience, along with the literature [34], tells us that not all customers will be able to make use of this advanced CDS functionality and achieve improvements in healthcare quality, safety or reductions in cost [35].

Another limitation is the fact that each of the systems is different, and the survey questions may over-emphasize or under-emphasize the unique capabilities of each system. However, the survey questions needed to be general and somewhat open-ended, to allow us to capture the broad aspects of what is available. After completing this exploratory analysis, future work may involve more specific questions targeted at a limited subset of products, in order to make better comparisons.

## Conclusions

Based on a relatively small sample of commercially-available electronic record systems, the clinical knowledge management capabilities of these systems were similar to those of the leading internally-developed systems. The majority of the vendor-supplied systems were capable of performing almost all of the key knowledge management functions we identified. If these well-designed systems are coupled with the other key socio-technical concepts required for safe and effective EHR implementation and use, we expect that the transformation of healthcare delivery processes that so many have predicted, is just around the corner.

## Competing interests

The authors declare that they have no competing interests.

## Authors' contributions

DFS and AW, designed, developed, tested and carried out the survey, analyzed the data, and wrote the paper.

SM - Assisted in carrying out the survey, organizing the data, and reviewed many drafts including the final version of the manuscript.

LS - Provided information for the survey and reviewed many drafts including the final version of the manuscript.

RSE - Provided information for the survey and reviewed many drafts including the final version of the manuscript.

WPN - Provided information for the survey and reviewed many drafts including the final version of the manuscript.

JSA - Obtained funding for the study, helped develop the survey questions and reviewed the final version of the manuscript.

BfM - Obtained funding for the study and reviewed many drafts including the final version of the manuscript.

All authors read and approved the final manuscript.

## Pre-publication history

The pre-publication history for this paper can be accessed here:

http://www.biomedcentral.com/1472-6947/11/13/prepub
